# Nutritional Knowledge, Confidence, Attitudes towards Nutritional Care and Nutrition Counselling Practice among General Practitioners

**DOI:** 10.3390/healthcare10112222

**Published:** 2022-11-07

**Authors:** Aleksandra Vrkatić, Maja Grujičić, Jelena Jovičić-Bata, Budimka Novaković

**Affiliations:** 1 Department of Pharmacy, Faculty of Medicine, University of Novi Sad, Hajduk Veljkova 3, 21000 Novi Sad, Serbia; 2 Department of General Education Subjects, Faculty of Medicine, University of Novi Sad, Hajduk Veljkova 3, 21000 Novi Sad, Serbia

**Keywords:** nutritional care, primary health care, physicians, non-communicable diseases, nutritional assessment

## Abstract

Nutritional care represents any practice provided by a health professional, aimed to improve the patient’s health outcomes by influencing patient’s dietary habits. Clearly, dietitians are the ones supposed to provide top-quality nutrition care, but their services are often inaccessible to many for various reasons. This obliges general practitioners (GPs) in primary health care to provide nutritional counselling to their patients to a certain extent. Preconditions to successful nutritional counselling are GPs with adequate nutritional knowledge, positive attitudes towards nutrition and nutritional care, self-confident and competent in nutritional counselling. Therefore, the aim of this review is to summarise currently available information on nutritional knowledge, confidence and attitudes towards nutritional care and nutrition counselling practice of GPs, as well as barriers towards provision of nutritional counselling. GPs do not consistently obtain satisfying results in nutrition knowledge assessments and their self-confidence in nutrition counselling skills varies. Studies suggest that nutritional counselling practice still has not met its full potential, and GPs frequently report various barriers that impair nutritional counselling practice. Thus, health policies that help overcome barriers and create stimulating environment for GPs to implement nutrition counselling strategies efficiently are the key to improving quality and quantity of nutritional counselling.

## 1. Introduction

Nutritional care represents any practice provided by a health professional, intended to improve the patient’s dietary habits and, consequently, health outcomes, especially in non-communicable diseases (NCDs) [1,2,3,4]. According to Nutrition Care Process Model approved by the Academy of Nutrition and Dietetics, nutritional care comprises nutritional assessment, diagnosis, intervention and monitoring and evaluation [5,6]. Nutritional assessment involves identification of the problem, based on systematically collected, detailed and relevant information related to patients’ nutrition [7,8]. If malnutrition is identified/diagnosed, the next step is appropriate nutritional intervention [7,8]. One aspect of nutritional intervention is nutritional counselling, defined as “a supportive process, characterised by a collaborative counsellor–client relationship, to establish food, nutrition and physical activity priorities, goals, and action plans that acknowledge and foster responsibility for self-care to treat an existing condition and promote health” [9]. Similar concept is proposed by The British Dietetic Association, in which nutritional practice includes six main steps: assessment, nutrition and dietetic diagnosis, strategy, implementation, monitor and review, evaluation [10].

General practitioners (GPs) in primary care setting are in an ideal position to counsel patients because they frequently interact with them and are familiar with their social environment and medical history [11,12]. Patients expect to get nutritional advice from GPs [13] as they consider GPs as the most adequate, reliable and approachable sources of nutritional information [11,12,13,14,15]. Therefore, GPs have an opportunity to positively influence patients’ behaviour and lifestyle habits [16,17,18,19,20]. Since their advice is held in high regard, GPs counselling activities have the potential to abate the incidence of preventable chronic diseases [21,22] and require good GP–patient communication and support in order to apply the recommendations rather than just being told (not) to do something, because the latter can provoke patients’ resistance [23]. Since GPs are the initial point in most patient’s healthcare, they should be well-equipped to perform adequate nutritional counselling, and moreover, to recognise whom and when to refer to a more specialised consultation with a registered dietitian [2]. Dietitians/nutritionists are healthcare providers whose services have the potential to improve patients health outcomes through a range of activities (i.e., nutrition counselling) that are supposed to affect the patients’ food and lifestyle choices, promoting health and managing diseases [24,25,26]. In the process, they have to make use of their unique knowledge, exhibit high levels of competence [26], and, finally, they ought to be skilled communicators of nutrition information [27,28]. But, healthcare professionals (HCP) need to work together and coordinate their efforts with their patients’ best interest in mind [24]. GPs and dietitians should collaborate, respecting each other’s unique knowledge, skills, and scope of practice, and understanding each other’s role in providing nutrition services [24], in order to build the needed respect between them, as essential members of interdisciplinary teams. But even when dietitians are available, health insurance coverage for dietitians’ visits varies greatly across different healthcare systems, from full coverage to none [29]. There is evidence that patients are less likely to use healthcare services not covered by their health insurance (e.g., dietitian services) [29], suggesting that they will turn to their GPs for nutrition guidance rather than cover the costs of specialised services out-of-pocket. In addition, GPs are reluctant to refer patients to services not included in their insurance plan [30,31]. Hence, it is important for GPs to be competent enough to perform adequate nutritional counselling, and moreover, recognise whom and when to refer to a more specialised consultation with a registered dietitian, or other specialised health professionals trained to provide nutritional care [2].

Despite the opportunities and benefits that nutritional counselling interventions can bring, the rate of its provision to patients remains low [32].

Unfortunately, nutrition education and training provided to healthcare professionals throughout their graduate and postgraduate programs are frequently deemed insufficient and inadequate, resulting in GPs’ limited knowledge and lack of confidence to provide nutrition care [33,34]. Some GPs further their nutrition knowledge and training through different programs, e.g., continuing medical education (CME) courses [35,36]. It has been shown that a high-quality, nutrition-related CME can boost doctors’ knowledge, skills and positive attitudes towards nutritional care, thus empowering them with greater confidence in own skills needed for nutritional interventions [35,36].

**Figure 1:**
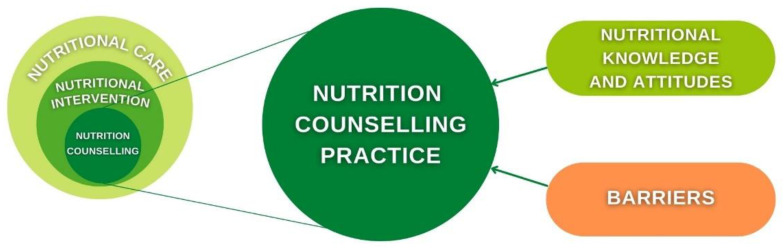
Nutrition counselling as a part of nutrition care and factors influencing nutrition counselling practice.

Success of nutrition counselling practice is dependent on GPs nutritional knowledge and confidence, their attitudes towards the significance of nutrition and nutrition counselling and existing barriers in practice [37,38] (Figure 1).Therefore, the aim of this review is to summarise currently available information on nutritional knowledge, confidence and attitudes towards nutritional care and nutritional counselling practice of GPs, as well as barriers towards provision of nutritional counselling.

## 2. Materials and Methods

A literature search was performed between 1 October 2021 and 1 January 2022, by searching PubMed, SCOPUS and Google Scholar databases. Databases search using combinations of keywords was done for all English language articles available in full published from 2008 to 31 December 2021. An additional search was performed on 31 May 2022, to check if there are any suitable scientific papers not previously included in the review. For the databases search, the primary entered terms and their combinations included the following: “nutrition”, “diet”, “lifestyle”, “nutrition care”, “knowledge”, “confidence”, “skills”, “attitudes”, “counselling”, “practice”, “general practitioners”, “physicians”, “barriers”, “primary health care”, in a form of both free text and MeSH terms. Original scientific and review articles yielded by the search that were available in full text and written in English were included in the assessment process. After elimination of duplicates, the remaining studies’ relevance to the research topic was evaluated based on their titles and abstracts by two independent researchers (AV and MG). Cases of disagreement concerning eligibility were resolved by discussion between the reviewers and decided in line with the reached consensus (AV, MG, JJB and BN). Those deemed unrelated to the study aim were excluded from further analysis. In all, 115 studies were included.

## 3. Nutritional Knowledge and Confidence in Nutrition Counselling Skills

### 3.1. Nutritional Knowledge

Nutritional knowledge is defined as the ability to identify basic facts about food and nutrients and their impact on one’s health [39]. Being equipped with the appropriate knowledge and skills for the assessment of nutritional risks enables GPs to take steps towards prevention, control and treatment of nutrition-related diseases [40]. However, research shows that GPs acquired different amount of knowledge through education and practice [41,42]. A study from Saudi Arabia [41] investigated GPs’ nutritional knowledge and nutrition management practice. It suggested that the mean percentage of correct answers to nutritional knowledge assessment questions is 50%, with 62% of GPs scoring lower than 50% and surprisingly, only 2% of GPs scoring more than 75% [41]. Highly variable percentages of correct answers indicate that there is an inconsistency in the level of nutritional knowledge among participants [41]. Results of a nutrition knowledge test performed in Qatar [43], reported the mean score for correctly answered questions of 64%. Similarly, HCPs, including primary care doctors, taking part in studies from Kuwait [44] and the USA [45] obtained 60% and 67% correct answers in nutritional knowledge assessment, which represents a moderate to good results, with US doctors achieving statistically significantly higher scores after completing certain CME programmes in nutrition (74% correct answers) [45]. Results obtained in another US study, indicate that primary care doctors answered correctly to 70% of the questions [46]. A survey conducted among GPs in Croatia [42] shows that the median of number of correct answers to the questions for nutrition knowledge assessment was 4 (range 0–10), and only 36% of participants reached satisfactory results (5 or more correct answers). In contrast, self-perceived nutritional knowledge is rated higher than the objective estimates [47]. Among GPs in Lebanon [48], 80% of GPs rated their nutritional knowledge as good or very good. The reason for this discrepancy might be the misinformation and misconceptions regarding lifestyle modification, including nutrition [47].

Lack of nutrition training and education can have a vital impact on the extent of nutrition information provided to patients. Moreover, a more dangerous perspective appears: insufficient nutritional knowledge can hinder the safety and efficiency of nutritional counselling, therefrom endangering patients. This stresses the urgency for reforms in medical education and training.

#### 3.1.1. Familiarity with Specific Areas of Nutrition and Factors Associated with Levels of Nutritional Knowledge

More detailed analysis of the nutrition knowledge assessment studies from Croatia [42], Qatar [43] and Saudi Arabia [49] shows that majority of GPs answered correctly the questions about the role of omega-3 fatty acids in the prevention of thrombosis [42,43], nutrition-related neural tube defects [42,43,49] and BMI values indicating obesity [42,43]. On the other hand, it seems that GPs are less familiar with the most concentrated food sources of vitamin B12 and type of fatty acids which are predominant in hydrogenated fat [49], eggs [43] and olive oil [42,49]. GPs demonstrated poorer knowledge regarding the effect of alcohol and dietary fibres on blood cholesterol levels, as well as food with the lowest glycemic index [43,49]. In addition, recommended number of fruit and vegetable servings per day, daily added sugar limit [50], nutritional assessment and obesity and nutrition in endocrine and cardiovascular diseases [51] are the areas in which it seems that the United States (US(A)) doctors lack in knowledge. Authors point out that further strategies for the improvement of GPs nutritional knowledge are needed [42].

No statistically significant difference in nutritional knowledge overall score was observed in relation to the age of GPs [42,44]. In a study from Qatar [43], male GPs achieved higher knowledge level about nutrition, whereas in a Croatian study [42], female GPs demonstrated better nutritional knowledge, with the authors pointing that this may be due to women being more interested in their diet and health than men. Opposed to these two studies, a study from Kuwait [44] found no significant difference in nutritional knowledge regarding the gender for most of the questions. Acquired specialisation was associated with better nutrition knowledge score in Croatian study [42], unlike in Qatar study [43], where no statistical difference was found. Higher scores were also obtained by GPs who took additional education/CME in nutrition [42,52]. Interestingly, GPs not suffering from nutrition-related chronic diseases achieved higher nutrition knowledge score, which implies that GPs suffering from these diseases are still not aware of the significant contribution of nutrition to their health [42]. Zeldman and Andrade [39] concluded that higher nutrition knowledge score was associated with age, gender, specialisation, years of experience, additional nutritional education and/or training of GPs. Some discrepancies were identified for age and gender, and some of the results did not confirm the connection between demographics and the level of nutritional knowledge at all [39]. This suggests that age and gender, with other mediators, may altogether be associated with level of nutritional knowledge [39]. As a consequence, it is difficult to draw conclusions on direct association when they are observed separately [39].

#### 3.1.2. Possible Strategies for the Enhancement of Nutritional Knowledge

Authors point out that the existing shortcomings in medical practice arise from insufficient medical knowledge and training on both undergraduate and graduate level [53], with medical curricula still not offering enough knowledge and training for contemporary nutritional counselling demands [54]. Medical school curricula not keeping up with the changes happening in nutrition-related disease demographics may eventually lead to the deficit of properly trained trainers available to train future practitioners [55]. Hence, one approach for the improvement of current GPs nutritional knowledge would be modifying medical school curricula in a way that equips future GPs with necessary knowledge and practical skills. For graduated doctors, the same should be done through CME, in order to fill the existing knowledge shortcomings, which prevent medical doctors from exercising nutritional counselling in practice. But the key to consider nutritional counselling more seriously and for GPs to engage in education and training in the field of nutrition involves adapting health policy measures in a way which motivates GPs to expand their knowledge.

When considering medical curricula across countries, a review by Crowley et al. [56] showed that in the USA, Canada, the United Kingdom (UK), Australia, and New Zealand there are externally visible curriculum guidelines for undergraduate nutritional education in medical schools, unlike in Ireland. Those in the USA are very detailed and prescription-oriented, whereas guidelines in Ireland do not specify nutritional recommendations [56]. According to the guidelines in Australia and New Zealand, all medical graduates should be able to identify nutritional risk, deficits and excesses, whilst in the UK and the USA only body weight assessment skills are emphasised, therefore potentially leading to exclusion of other important aspects of nutritional assessment and nutritional care in general [56]. In 2015, only UK had mandatory nutritional guidelines for curriculum in medical schools [56]. Presented differences in curricular guidelines on nutrition altogether pose an obstacle to reaching a uniform curriculum and nutritional counselling practice, considering that these countries share the language, and some of the food items [56]. These countries are some of the countries that eventually applied the “Need for Nutrition Education Project” or the NNedPro approach [57]. The NNEdPro approach proposes and evaluates interventions in nutritional education, and presents a good example of improvement of nutritional education. Some of the core principles of the intervention involved highlighting the importance of nutrition as a part of a doctor’s responsibilities, and appreciation of the scope of clinical and public health nutrition [57]. NNEdPro eventually resulted in reinforced cross-border exchange of information and knowledge, cooperation and consensus on best practices in nutrition medical education. Another great, instructive example of the integration of nutrition into medical curriculum is a course on University of Crete, Greece [55]. It was a dedicated course for third-year students, consisting of 6 h of lectures and 25 h of practical sessions [55]. In this course, students are involved both as counsellors and patients in nutritional assessment. Similar concept was applied in Israel, regarding nutrition, exercise and lifestyle behaviours [55].

Nutritional knowledge and nutrition counselling skills can be improved through CME, in which USA is considered the leading country in MD’s training in nutrition [55]. Several of the most successful programmes include, for example, Lifestyle Medicine (course offering practical skills in counselling and culinary education tools and strategies), Mayo Clinic Nutrition and Wellness in Health and Disease (dealing with ambulatory lifestyle-related topics), Nutrition & Health Conference (reviews the latest nutrition and health related information) [58]. Moreover, workshops held in the USA [35] and Greece [52] offered information, tools and technical assistance for counselling in nutrition-related diseases and resulted in higher self-reported knowledge and confidence in primary care professional’s (including GPs) counselling skills.

This brief summary with examples of nutritional education and training of medical students and doctors across countries shows that certain steps forward have been made in this field. Yet, it reveals that nutritional education is still not standardised across different countries, leaving space for future suggestions and considerations on whether standardisation is needed, to what extent it is feasible and what is the best solution.

#### 3.1.3. Confidence in Nutrition Counselling Skills and the Link between Knowledge and Confidence

Significance of nutritional knowledge and translation of knowledge to action is highlighted by the fact that insufficient knowledge can often compromise confidence in nutrition counselling skills [59,60]. GPs confidence further influences the provision of nutritional care [61,62], thus representing one of the main steps to successful counselling.

A survey conducted among GPs in Australia [40] shows that 95% of them stated feeling at least “somewhat confident” in providing nutritional information to the patients. A Canadian survey [63] demonstrates that nearly 90% of family medical doctors (MDs) are comfortable with their counselling skills about healthy nutrition, although significantly lower percentage (around 40%) of them actually provide nutritional counselling always or often. In some of the surveys [64,65,66], GPs expressed doubts about their own and their colleagues’ preparedness for nutritional counselling. Slightly more than 10% of internal medicine residents from Switzerland feel that MDs are adequately trained in clinical nutrition [65]. Australian GPs claim to have limited confidence in their own knowledge and abilities regarding nutritional counselling [64]. In favour of emphasising the benefits of higher confidence regarding nutritional counselling, Znyk et al. [66] showed that GPs who have more confidence in their knowledge, and those working in public sector, are more likely to provide lifestyle recommendations (including the ones related to nutrition) to patients who have no chronic lifestyle-related disease, but are following an unhealthy lifestyle.

#### 3.1.4. Confidence in Counselling Skills regarding Specific Areas of Nutrition and Factors Associated with Confidence in Nutrition Counselling Skills

Confidence of GPs regarding provision of nutritional care differed depending on the disease and patient subgroup [61,65]. GPs felt confident when counselling patients about nutrition in weight loss, diabetes, cardiovascular risk and osteoporosis, while being less confident in nutritional counselling regarding cancer prevention, weight loss as a consequence of chronic illness and sarcopenia [61,65]. They are more confident in providing nutritional care to elderly patients and pregnant/breastfeeding women, then to infants’ and toddler’s parents/caregivers [61], therefore pinpointing the population groups to which more attention should be paid in nutritional education and training.

#### 3.1.5. Confidence vs. Self-Efficacy

Self-confidence reflects strength of belief, but not the target or specific domain for that belief [67]. Self-efficacy, in contrast, is defined as the “confidence to carry out the courses of action necessary to accomplish desired goals” and reflects internal personal beliefs, which are goal-oriented, context-specific and future-oriented [67]. Smith et al. [37] assessed self-efficacy of senior medical residents from Ohio indicating that senior residents perceived lower self-efficacy in their own abilities regarding nutrition and obesity counselling.

#### 3.1.6. Factors Associated with Self-Efficacy in Nutrition Counselling Skills

Gender (female GPs report significantly lower self-efficacy), age (older GPs claim more self-efficacy), medical school curricula and CME dedicated to nutrition, acquired specialty (and specifically, family medicine residency compared with internal medicine and gynaecology residency), ambulatory placement during residency (residents who spent more time in ambulatory placement), country where the training was obtained (US-trained residents reported significantly lower self-efficacy) are all associated with self-efficacy to provide nutritional and obesity counselling [37,59], supporting the view that more engagement in nutritional counselling through education and practice boosts self-efficacy.

Given that there still are certain areas in nutrition in which GPs do not feel entirely trained and confident to provide nutritional counselling, future changes in medical education, training and health policies should aim to empower GPs with greater confidence, which will eventually lead to building well-equipped, competent health care professionals.

Factors associated with GPs levels of nutritional knowledge, confidence and self-efficacy in nutrition counselling skills are summarised in Table 1.

## 4. Attitudes towards the Significance of Nutrition and towards Nutritional Counselling

Favourable attitudes towards the importance of nutrition and nutritional counselling positively influence the provision of nutritional care [59,60].

### 4.1. Attitudes towards Significance of Nutrition

In general, GPs attitudes towards the role of nutrition in prevention and treatment of disease are positive [59,60]. Over 90% of Canadian GPs agreed with the aforementioned claim, in relation to chronic diseases [68]. Majority of the surveyed US GPs concurred that counselling on healthy diet would improve patient health outcomes [50,69], such as their Australian colleagues [64]. Australian medical educators [70] agreed on nutrition being important, but rather superficially approached area of healthcare in general practice.

### 4.2. Attitudes towards Significance of Nutritional Counselling

More than 90% of Lebanese GPs agreed that nutritional counselling is effective in changing patient behaviour [48], though not that many Canadian GPs strongly endorsed that viewpoint (roughly 55% somewhat agreed and 20% agreed) [68]. Additionally, the majority of GPs registrars from Australia [40] recognised the importance of giving their patients basic nutrition and lifestyle advice. Han et al. [65] found that 70% of internal medicine residents from Switzerland believed that all doctors should be able to provide nutritional assessment. A survey by Crowley et al. [61] reported very high interest of Australian GPs towards provision of nutritional care.

Dumic et al. [1] examined Croatian GPs attitudes toward both nutrition and nutritional care. Only 36% of GPs achieved a satisfactory number of positive attitudes (5 or more out of 10) concerning the importance of nutrition in prevention and treatment of chronic diseases and nutritional care (median number 3, range 3 to 9) [1]. Even though just a third of respondents expressed positive attitudes towards nutrition and nutritional care, the majority claimed providing nutritional care in everyday practice [1]. This may imply that positive attitudes are not the only motivating factors for the actual provision of nutritional counselling.

These findings might be explained by the fact that the importance of nutrition for health, as well as the role of health professionals in nutrition counselling have been heavily promoted during the past decade or two. Nutrition has become the hot topic of many continuing education programmes, congresses, international meetings and this was further solidified by the promotion of healthy lifestyle, including nutrition, via different social media during the same period.

### 4.3. Factors Associated with Positive Attitudes towards Significance of Nutrition and towards Nutritional Counselling

It was noted that the Croatian GPs who gained additional education in nutrition or those who did not suffer from nutrition-related chronic diseases had more positive attitudes towards both significance of nutrition in chronic disease prevention and treatment and nutritional care [1], suggesting that additional education may raise more interest and awareness towards them [2,71]. There was no significant difference between GPs in previously described attitudes concerning gender, age, years of experience nor obtained family medicine specialisation or without it [1], although Wynn et al. [68] reported that younger Canadian GPs have more positive attitudes toward nutrition. The positive attitudes might be related to the campaigns and programmes that were set in the past decades, raising awareness of the importance of nutrition in well-being and disease, as well as the importance and the role of healthcare professionals in nutritional counselling.

Factors associated with general practitioners’ attitudes towards significance of nutrition and towards nutritional counselling are summarised in Table 2.

### 4.4. GPs Perception of Duties regarding Nutritional Counselling

GPs perception of responsibility and duty is of great concern for efficient provision of nutritional counselling and nutritional care in general [70]. Some of the GPs consider nutritional counselling as a part of their regular medical practice [48,51], since they understand that it can encourage positive changes towards appropriate dietary behaviour in patients [64]. Although agreeing with this claim, 60% of Australian GPs did not consider a more detailed discussion on nutritional topics as a part of their job [61]. Studies conducted in Germany and the USA are in favour of the rather positive attitudes regarding physicians role in health promotion, disease prevention and obesity counselling [12,72,73]. However, studies from Croatia [1], Saudi Arabia [41], Australia and New Zealand [70] suggest that GPs express modest interest in nutritional care, which might be the consequence of insufficient nutritional knowledge and lack of suitable education and training [41]. According to Ball et al. [70], the gap between considering nutritional assessment and interventions as a priority in general practice, and actual insufficient inclusion of nutritional approach in everyday practice was observed. It is not clear whether limited effectiveness of Australian and New Zealand GPs in this field come from the lack of competency or other barriers or effectiveness of dietary intervention itself [70]. Similar findings were observed in a study conducted in the USA [51], which shows that regardless of having positive attitudes about nutritional care, interns do not feel proficient enough to provide adequate counselling, with 86% of them agreeing that most physicians are not trained to discuss nutrition with patients. Rather negative perception towards nutrition being a doctor’s responsibility is still present among some GPs [55,74]. As most of the aspects of nutritional counselling, perception of duty in this area most likely has a complex background, based on previous knowledge and training in nutrition, barriers related to healthcare system, and not to neglect personal beliefs and prior personal and professional experience.

Apart from GPs attitude toward their duty, it is reasonable to take into account how patient’s behaviour affects GPs willingness to discuss nutrition [75,76]. Some of the interviewed primary care doctors feel that most patients do not understand the importance of dietetic referral, whereas some GPs think that the importance of making lifestyle changes is understood over time [75], and those GPs impressions can greatly influence counselling provision. This aspect of motivation to discuss nutrition might also be in relation with GPs personality.

In EUROPREVIEW study [76], 90% of participating patients from 22 European countries endorsed the importance of changing unhealthy dietary habits, more than 70% of those following an unhealthy diet were aware of the need for improvement, and 50% of them would like to receive advice from a GP. As with GPs, expressing positive attitudes does not necessarily convene into particular lifestyle change in patients, but it represents a good starting point and an encouragement for healthcare professionals to practise nutritional counselling.

In order to raise awareness of nutrition as the key to prevention and treatment of chronic diseases, effective strategies to improve GPs current attitudes regarding the subject should be developed and implemented. But this calls for an orchestrated effort of all health-related policymakers directed at creating a healthcare system with identified and minimised barriers and precise descriptions of responsibilities of every member of a multi-disciplinary team in order to provide effective nutrition counselling to patients.

## 5. Nutrition Counselling Practice in Daily Work with Patients

Today’s imperative is that all health professionals should be competent to practise evidence-based nutritional counselling to at least a minimal extent [77], as even a basic advice might play an important role in changing health behaviour of a patient [78]. However, GPs are not always able to translate knowledge into practice and provide adequate nutritional advice, an advice that could possibly lead to significant changes in the patient’s diet [77].

### 5.1. Frequency, Trend and Specifics of Nutrition Counselling Practice

About 70% of primary care MDs in a German study [12] stated they routinely provide brief consultations on diet modification to at least one half of their patients. Almost all of the surveyed Croatian [79] and Lebanese GPs [48] claimed that they provide nutritional support to their patients in everyday practice. However, less than 20% of GPs who participated in a Croatian study [79] stated they provide counselling to all patients, while 80% of them provided nutritional counselling exclusively to patients with specific health risks. Forty-three percent of surveyed Australian patients with type 2 diabetes reported they received nutritional care from their GP, whereas about a third stated that provided nutritional care was effective in changing their nutritional behaviour [80]. A study [59] showed that a third of Saudi Arabia primary care MDs reported “never” or “rarely” counselling patients on nutrition during the past month, while a third did counselling only “half of the time”. In an Australian study [61], over 90% of GPs reported proactively discussing the topic of nutrition with patients, while almost 80% also stated that patients initiated the discussion. In general, with limited time per visit, it would be hard to provide adequate nutritional counselling, bearing in mind the other demands GPs are faced with in their daily practice. However, it would be of great importance that GPs keep in mind the saying “Prevention is better than cure” whenever the circumstances are favourable.

A longitudinal Dutch study [11] describes that the GPs interest in the effect of nutrition on health increased from 1992 to 2007, but the frequency of nutritional counselling on daily basis declined. Decline in nutritional counselling was also observed among GPs in the US studies for 1995–2007 [81] and 2005–2015 period [82]. A specific trend of nutrition counselling rates among Lithuanian GPs from 2000 to 2014 is [78]: counselling on nutritional habits was provided to 23% of the overweight/obese patients in 2000, to 37% in 2010, and then dropped to 30% in 2014. The observed trends show that the rate of provision and frequency of nutritional care in many countries is still suboptimal [11,78,81,82]. In fact, it appears that the rates of nutritional counselling in the US continue to decrease from 1995 onwards, which might be caused by challenges and additional demands in everyday practice experienced by GPs (filling in electronic health records, managing the growing number of patients with chronic disease, fulfilling requirements for the overall improvement of quality of care), sometimes even leading them to frustration [81]. Moreover, decreasing trend might be a reflection of US GPs attitude and self-efficacy regarding nutritional care, or a consequence of the implemented health policies [82]. These specific results were obtained in similar, but not identical periods and might have been affected by country-specific circumstances. Therefore, any uni-dimensional conclusion drawn from them should be interpreted cautiously.

From 1992 to 2007, Dutch GPs’ perception of health education and prevention did not change, while daily activities moved from curative towards preventive [11], whereas primary care professionals from the USA [83] and Canada [75] reported that GPs usually focus on pathophysiology of the illness and disease management (e.g., applying objective anthropometric measures) rather than conducting nutritional assessment, even for patients with weight-related chronic diseases. As a consequence, the importance of body weight is overemphasised by GPs, while the focus should be on nutritional assessment [75,84]. When doctors engaged in direct discussion on nutrition, they usually discussed ill effects of diet high in sugar, sodium and/or fried food, impact of fat and cholesterol on health [83], instead of practising a more wholesome approach to overall nutrition.

### 5.2. GPs Perception of Own Responsibility in Nutrition Care Process and Practice regarding Dietitian Referral

Evidence suggest that majority of GPs shared the opinion that counselling patients on nutrition is their responsibility [51]. On the other hand, some of the GPs consider weight reduction as patient’s responsibility, or themselves as passive supervisors [85], possibly being discouraged by their previous negative experiences with. They may have been involved in unsuccessful counselling with unrealised goals, but it should be noted that GPs are not the only factor associated with the achievement of desired behavioural change in a patient [11,74,86]. However, this should not be a reason strong enough for GPs to quit counselling and motivating patients to change their diet [65,66]. Different viewpoints among GPs regarding their role in nutritional care may stem from ambiguous definitions of their duties.

Referring patients to registered dietitians could be one of the crucial steps in helping patients in need of nutritional care and nutrition counselling to more clearly understand and get sufficiently motivated to implement suggested behavioural changes as a new lifestyle [87,88]. Beyond the significance of individual efforts of HCPs, it has been shown that team-based cooperation between HCPs from different fields of expertise (e.g., dietitians/nutritionists, GPs) enhances patients’ education and improves preventive and therapeutic outcomes [26,89,90]. It reduces the costs of healthcare services and as a consequence of delegation of the tasks and provides GPs with more time for other patient services [26,90]. If interprofessional collaboration is to be successful, team member duties must be well-defined and understood, communication and knowledge exchange between team members should be efficient [26,90]. Unfortunately, at this point in time, the border between GPs’ and dietitians’ responsibilities within the healthcare systems is often blurred, making this an issue that should be prioritised and resolved in the near future [26]. Smith et al. [84] showed that only a few US primary care MDs reported continual referral of their patients for further management on nutrition and systematic tracking of patient behaviour. In an Australian study [91], just 0.26% diagnoses resulted in referral of a patient to a dietitian/nutritionist during 2010–2015. Although 95% of GPs from a Canadian study [68] stated they redirected patients to dietitians, there was no association between the frequency of referrals and the number of patients for whom they believed were in need of counselling, nor for those patients who received counselling. Financial difficulties and healthcare system limitations (waiting time; lack of available, on-site dietitians) might be the cause of insufficient referral [48,75]. However, some studies bring evidence in support of GPs increasing contribution to more intensive referral practice with over 60% of GPs claiming they redirect patients to dietitians [83], and similar percentage claiming they are always or often redirecting [63]. Additionally, GPs from rural areas significantly more frequently referred patients to dietitians, which may have been the result of working in a smaller health centre, where different HCPs cooperate more [68]. Again, certain barriers may compromise the frequency of referral to dietitians, but patients should be referred whenever that is in their best interest, keeping in mind that the utilisation of dietitians’ services is dependent on insurance coverage.

### 5.3. Factors Influencing GP’s Decision to Provide Nutritional Counselling

In the light of sociological factors influencing physicians’ decision to provide nutritional counselling, Eisenberg set up a model, consisting of four groups of factors: physician-related factors, patient-related factors, the physician–patient relationship and physician’s interaction with healthcare systems [82,92].

Regarding the physician-related factors influencing provision of nutritional care to patients, it was found that physicians in primary care setting were more likely to provide any type of counselling than non-primary care physicians, potentially on account of more frequently meeting patients who would benefit from this type of counselling [82]. It was also demonstrated that female GPs were more eager to provide nutritional counselling [59,72,81]. This is oftentimes ascribed to the female communication style, usually involving more empathy, interest in patient’s experience with illness and psychological component of it and engagement in preventive service implementation [93]. Predicting and motivating factors for provision of nutritional care also included: higher self-assessment of GPs knowledge in the field nutritional science [48], confidence in counselling about nutrition [11,59,68], previous or additional nutrition education [1,59], higher professional qualification [59], GPs considering nutritional counselling as their responsibility [48,68] and having positive attitude towards the effectiveness of counselling on patient’s behaviour [68]. These findings support the premise that it is important to invest in education, training and develop positive attitudes towards nutritional care, in order to increase the chances of GPs proactively implementing nutritional care into practice [40,64]. Furthermore, another physician-related factor that can influence the provision of nutritional care is physician’s attitude towards overweight and obese patients [94,95]. Some GPs shared the attitudes that overweight and obese patients are lazy, undisciplined, without motivation and willingness to change, which, in turn, affected GPs motivation to provide counselling, leading to suboptimal care [85,94]. Although attitudes of HCPs towards obese patients were negative [94,95], they have improved over time and HCPs decision on providing nutritional care and its quality were not as biased by HCPs attitudes as in the past [94]. This is important, because overcoming the stigma around obese patients can improve the frequency and extent of nutritional counselling. Moreover, unstigmatised patients may show more motivation and readiness to change their previous unhealthy behaviour.

When considering patient-related factors, it has been demonstrated that older patients were less likely to receive behavioural counselling [82]. Males had a higher probability to receive nutritional counselling [78]. Ahmed et al. [32] came to opposing conclusions regarding the gender differences. Patients’ weight was also a factor affecting the GPs decision to provide counselling [32,78,82,96]. Obesity was one of the most significant predicting factors for provision of nutritional counselling [96]. Underweight [82] and overweight patients [32,82], obese patients at higher risk [96] and/or diagnosed with more chronic conditions [78], were more likely to receive nutritional counselling than patients with normal body weight or without risk and chronic disease. Patients with chronic disease were more likely to be counselled, in comparison to patients with new health issues [75,78,82]. Repeatedly, GPs seem to be focused more on patients with the diagnosed disease, missing the chance to prevent nutrition-related diseases in other patients [97]. This decision might stem from time limitations in daily work with patients which make GPs more dedicated to resolving acute problems [97]. It might also be that GPs are more likely to counsel high risk, chronic disease patients because they see them as a demographic that is more motivated to implement their GPs advice and change their unhealthy habits. When it comes to older vs. younger patients, it is possible that GPs decide against counselling the older patients believing they are not as willing to change their longstanding habits as the young. Additionally, older patients cognitive and mental capacity might be affected by their health issues or medications, reducing their chances to follow nutritional instructions consistently.

Among the physician–patient relationship factors influencing GPs willingness to provide counselling, it is of substantial importance for GPs to have good communication skills, because the success of nutritional counselling can greatly depend on the quality of communication and the relationship built with the patient [98,99]. In addition, routine visits to GPs [78,82] and longer lasting patient visits also contribute to a higher likelihood of patients being provided with nutritional counselling [96], because more time spent with the patient enables more thorough counselling and, paired with good communication skills, builds a trustworthy relationship [66,100]. Apart from getting polished by experience, communication skills can be improved by additional training, suggesting that this area of a physician–patient relationship can be significantly enhanced.

Considering physician–healthcare system interaction characteristics, it has been shown that patients in non-metropolitan areas were less likely to receive nutritional counselling from GPs [82], but at the same time, were more likely to be referred to a dietitian, possibly because in smaller medical centres GPs interact and communicate more with other HCPs [68].

In order to improve current nutrition counselling practice, new health policies should be implemented and stimulating environment created for GPs, in which they would feel empowered and aware of the potential benefits of counselling they would routinely provide [2]. Factors known to influence nutrition counselling practice should be carefully considered during the creation of policies. In addition, policies should be tailored to suit country-specific needs.

Factors influencing general practitioners’ decision to provide nutritional counselling are summarised in Table 3.

### 5.4. Barriers to Provision of Nutritional Counselling by GPs

Lately, a trend of decline in lifestyle/nutritional counselling has been noted [78] despite the rising prevalence of overweight and obesity, possibly due to different barriers being experienced by GPs [64] (Table 4). Some of the frequently reported barriers to nutritional counselling include time constraints, lack of nutritional education and lack of patients’ adherence to the advised diet plan [48,77].

GPs often quote time constraints as a major barrier in nutritional counselling [64,72,101,102]. Administrative workload contributed to GPs lack of time for nutritional counselling [66]. A study conducted in Saudi Arabia [59] showed that almost 60% of GPs claimed they spent less than 3 min providing nutrition care advice, and the same amount of time the USA GPs [62] spent on counselling patients about diet and lifestyle to prevent cardiovascular diseases during routine appointments. Patient visits to female GPs lasted for almost 2.5 min longer than to male GPs, although the results of a meta-analysis have shown great variations [103]. The observed difference might be explained by male GPs having more patients, leaving them less time for counselling [72]. Considering that Parker et al. [47] stated that the entire patient visit lasts approximately 5–7 min and includes everything from examination to re-issuing medical prescriptions and counselling, it is clear that a restriction of 3 min for counselling can significantly harm its quality. According to Rao et al. [104], in 2015, an average duration of patient visit in the USA was 21 min, potentially opening the space for longer, comprehensive nutritional counselling. When majority of GPs are provided with no more than several minutes per patient per visit, it does not come as a surprise that time constraints are often quoted as barriers in surveys concerning nutritional counselling.

Many GPs reported lack of nutritional education [70,83,105], knowledge [102,106,107], training [69,73] and skills [40,108] as some of the important barriers in nutritional counselling. Data show that existing undergraduate and postgraduate training does not fulfil current requirements in nutritional care [65]. It has been noted that the US medical schools offer different amounts of nutritional education and training [77]. Consequently, lack of knowledge and skills leads to lack of expertise and/or confidence in diagnosing nutrition-related diseases and provision of proper dietary advice which, in turn, makes the GPs deal with health consequences of malnutrition instead of prevention [55,60,65,83]. Research shows [61] that GPs are willing to overcome the aforementioned barriers as they did express in more nutritional education through CME. Thus, high-quality education is the key for overcoming the issue concerning graduated, experienced GPs.

There are also studies which point out GPs negative perception of the importance of nutrition [102] and low interest in nutrition as obstacles towards providing adequate nutritional care [109,110]. The raising concern among GPs that the usually recommended preventive measures may not lead to expected outcomes in health promotion and chronic disease prevention was also noted [65,66,108]. But it has been pointed out that gaining more knowledge on the effects of nutrition on health outcomes makes GPs more likely to refer their patients to dietitians/nutritionists [111]. Therefore, educational programmes with real-life examples of successful nutritional interventions can significantly contribute to overcoming this barrier.

Healthcare professionals still have not reached a unanimous opinion regarding the precise scope of responsibilities of health care professionals in approaching nutritional counselling [70,108]. Some authors cited that there is a prevailing attitude that nutritional interventions are not primarily doctors’ responsibility, but rather of other HCPs [55]. Inadequate cooperation with other HCPs have the potential to undermine effective lifestyle/nutritional counselling [72]. Therefore, good interdisciplinary cooperation of different HCPs should be an imperative [108], because it should result in the most comprehensive approach to patient care.

Patient’s lack of adherence to dietary advice [47,48,61,107] and motivation [60,85] to follow the advice are acknowledged as additional obstacles to successful nutritional counselling. In a Canadian study [23] patients quoted they do not want or like to follow the recommended diet-related behaviours, but also “not knowing it is important” and “not knowing it is recommended”, which adds another dimension to nutritional counselling barriers: uninformed patients. Unavailability of nutrition-related educational materials for patients (printed handouts, online handbooks, mobile applications, webinars, computer-guided instructions) may also be considered an obstacle to nutritional counselling [69,108]. Apart from the educational tools for patients, healthcare practitioners should have easy access to the resources that contain necessary information, objectives and guides for nutritional counselling (via Internet and smart devices, if possible) [108]. In the absence of these resources, nutritional counselling may not be optimal [108]. But motivating patients to change previous unhealthy dietary habits requires more than just a provision of nutritional information: it involves continuous, long-term support [77,100].

On the road to success, GPs should lead by their own example—“practising what they preach”, to earn respect from patients and increase the likelihood of their advice being implemented and followed by patients [55,112]. This is not always the case, since sometimes one can be a great counsellor to others, failing to pursue changing his own “bad habits”.

Additional obstacles in performing nutritional assessment and counselling are inadequate reimbursement and financial incentives [66,70,83,101,106,108]. In Germany, for example, preventive counselling that GPs provide is mostly reimbursed by private insurance companies, which means that patients who have private insurance get counselling, including the one on nutrition, more often [113]. From the financial aspect, insurance coverage of fees often compromised referrals to dietitians [108]. If these services are not covered by insurance, less primary care professionals will implement such measures into their everyday practice [32,72,82]. This barrier can be mitigated exclusively with the support of the government and other parties included in insurance systems.

In the light of the much needed systematic and organised approach to nutritional care, establishing guidelines and/or protocols have also been recognised by GPs as a way of advancing quality of care in both primary care and clinical practice [70,108]. But despite the existence of some official guidelines and programmes aimed to improve and increase the rates of nutritional counselling (US “Healthy people 2020” and the “European Food and Nutrition Action Plan 2015–2020”), the number of physicians following and implementing these recommendations is still low [114].

Cultural differences and language barriers may pose a barrier to achieving effective counselling [47]. This might be of higher importance in specific countries or regions that are more ethnically diverse or more affected by immigration.

Nonetheless, some of the GPs do not perceive any barriers, as it was the case in 8% of participating GPs from Croatia [79] or 18% of surveyed Brazilian non-dietitian health professionals, including GPs [107].

Regarding the factors influencing GP perception of barriers, male GPs are more likely to perceive barriers as important for their daily counselling practice, which may be explained by different subjective values towards nutrition and health between male and female GPs [72]. A conclusion can be made that female GPs are more motivated to bypass obstacles on the road to successful delivery of nutrition counselling services.

To optimise nutrition counselling practice, it is necessary to put in more effort to overcome the existing barriers [115]. If GPs are recognised and awarded by national health officials and provided with incentives and necessary resources, they would be more motivated to provide better care. These actions should be initiated by redirecting health policies towards primary prevention and health promotion in order to successfully implement nutritional counselling among GPs.

## 6. Strengths and Limitations of the Review

The strength of this review is that it presents a comprehensive overview of currently available information on GPs nutritional knowledge, their attitudes towards the importance of nutrition and nutritional counselling, confidence in own skills and own practice competences in nutritional counselling. It offers a wholesome insight into the current state of nutritional counselling provided by GPs, including the potential barriers that hinder its quality. Therefore, it might serve as a guide for stakeholders policymakers interested in bettering the provision of nutritional care.

The limitation of this review is that it is not a systematic review. The search for eligible articles was performed using three databases, namely PubMed, SCOPUS and Google Scholar. In addition, the search was limited to articles written in English. Consequently, some of the articles not included in the said databases or written in languages other than English that are relevant to the study might have been omitted. Moreover, some of the viewpoints expressed in this study might have been affected by the exclusion of non-English articles from the study, as the articles written in other languages likely contain culturally or ethnically specific aspects of nutritional counselling.

## 7. Conclusions

NCDs are a major burden for health care systems in the 21st century. They can be controlled, treated and most importantly, prevented with strategies comprising nutritional care. Although dietitians/nutritionists exist within the healthcare system, the specifics of primary care settings allow for GPs to communicate with patients easily, with strong emphasis on seeing the early signs of the disease and act preventively. Given that NCDs pandemics inevitably continues to grow, systematic efforts of policymakers should be dedicated to additional education and training, which will provide GPs with adequate level of knowledge, skills and confidence in their own nutrition counselling skills. Health policies must be carefully created in a way that develops positive attitudes towards nutrition and nutritional care, which will be transferred from GPs to patients. If health policies succeed in removing and minimising present barriers to nutritional counselling, the aforementioned strategies would motivate and encourage GPs to successfully implement nutritional counselling into practice and help combat NCDs and improve the overall health status of their patients.

## Figures and Tables

**Table 1:** Factors associated with general practitioners’ levels of nutritional knowledge, confidence and self-efficacy in nutrition counselling skills.

Factors Associated with General Practitioners’ Levels of Nutritional Knowledge	Factors Associated with General Practitioners’ Confidence in Nutrition Counselling Skills	Factors Associated with General Practitioners’ Self-Efficacy in Nutrition Counselling Skills
Gender ^1^	Type of patient’s diagnosis	Gender
Age ^1^	Patient subgroup	Age
Medical school curricula dedicated to nutrition		Medical school curricula dedicated to nutrition
Additional/continuing medical education dedicated to nutrition		Additional/continuing medical education dedicated to nutrition
Acquired specialisation ^1^		Acquired specialisation
Years of experience		Ambulatory placement during residency
Suffering from nutrition-related chronic diseases		Country in which the training was obtained

^1^ Some of the studies failed to confirm the connection between gender, age and acquired specialisation and general practitioners’ levels of nutritional knowledge [39,42,43,44].

**Table 2:** Factors associated with general practitioners’ attitudes towards significance of nutrition and towards nutritional counselling.

Factors Associated with General Practitioners’ Attitudes towards Significance of Nutrition and towards Nutritional Counselling
Gender
Age
Additional education in nutrition
Years of experience
Suffering from nutrition-related chronic diseases
Holding/Not-holding a specialist degree

**Table 3:** Factors influencing general practitioners’ decision to provide nutritional counselling.

Physician-Related Factors	Patient-Related Factors	Physician–Patient Relationship Factors	Physician–Healthcare System Interaction
Type of healthcare system (primary/non-primary)	Age	Communication skills	Type of settlement (urban/rural)
Gender	Gender	Frequency of patient’s visits to general practitioner	
Self-assessment regarding knowledge in the field nutritional science	Weight	Duration of patient’s visits to general practitioner	
Confidence in counselling about nutrition	Previous diagnosis of chronic disease/diseases with or without additional risk/s		
Previous or additional nutrition education			
Level of professional qualification			
Perception of responsibility considering nutritional counselling			
Attitude towards the effectiveness of counselling on patient’s behaviour			
Attitude towards overweight and obese patients			

**Table 4:** Barriers to provision of nutritional counselling perceived by general practitioners.

Barriers
Time constraints
Lack of nutritional education, knowledge, training and counselling skills
Negative perception of the importance of nutrition
Low interest in nutrition
Lacking definitions of the precise scope of responsibilities of health care professionals in approaching nutritional counselling
Patient’s lack of adherence to dietary advice and motivation to follow the advice
Unavailability of nutrition-related educational materials for patients
Limited access to the resources that contain necessary information, objectives and guides for nutritional counselling for healthcare practitioners
General practitioners not adhering to their own advice
Inadequate reimbursement and lack of financial incentives
Absence of suitable guidelines and/or protocols
Cultural differences and language barriers
Different subjective values towards nutrition and health between male and female general practitioners

## Data Availability

Not applicable.
